# Calcium and Potassium Supplementation Enhanced Growth, Osmolyte Secondary Metabolite Production, and Enzymatic Antioxidant Machinery in Cadmium-Exposed Chickpea (*Cicer arietinum* L.)

**DOI:** 10.3389/fpls.2016.00513

**Published:** 2016-04-27

**Authors:** Parvaiz Ahmad, Arafat A. Abdel Latef, Elsayed F. Abd_Allah, Abeer Hashem, Maryam Sarwat, Naser A. Anjum, Salih Gucel

**Affiliations:** ^1^Department of Botany and Microbiology, Faculty of Science, King Saud UniversityRiyadh, Saudi Arabia; ^2^Department of Botany, Sri Pratap CollegeSrinagar, India; ^3^Botany Department, Faculty of Science, South Valley UniversityQena, Egypt; ^4^Biology Department, College of Applied Medical Sciences, Taif UniversityTaif, Saudi Arabia; ^5^Department of Plant Production, Faculty of Food and Agricultural Sciences, King Saud UniversityRiyadh, Saudi Arabia; ^6^Mycology and Plant Disease Survey Department, Agriculture Research Center, Plant Pathology Research InstituteGiza, Egypt; ^7^Pharmaceutical Biotechnology, Amity Institute of Pharmacy, Amity UniversityUttar Pradesh, India; ^8^Department of Chemistry, Centre for Environmental and Marine Studies, University of AveiroAveiro, Portugal; ^9^Centre for Environmental Research, Near East UniversityLefkosa, Cyprus

**Keywords:** antioxidant enzymes, cadmium toxicity, chickpea, oxidative stress, organic solutes, secondary metabolites, yield attributes

## Abstract

This work examined the role of exogenously applied calcium (Ca; 50 mM) and potassium (K; 10 mM) (alone and in combination) in alleviating the negative effects of cadmium (Cd; 200 μM) on growth, biochemical attributes, secondary metabolites and yield of chickpea (*Cicer arietinum* L.). Cd stress significantly decreased the length and weight (fresh and dry) of shoot and root and yield attributes in terms of number of pods and seed yield (vs. control). Exhibition of decreases in chlorophyll (Chl) *a*, Chl *b*, and total Chl was also observed with Cd-exposure when compared to control. However, Cd-exposure led to an increase in the content of carotenoids. In contrast, the exogenous application of Ca and K individually as well as in combination minimized the extent of Cd-impact on previous traits. *C. arietinum* seedlings subjected to Cd treatment exhibited increased contents of organic solute (proline, Pro) and total protein; whereas, Ca and K-supplementation further enhanced the Pro and total protein content. Additionally, compared to control, Cd-exposure also caused elevation in the contents of oxidative stress markers (hydrogen peroxidase, H_2_O_2_; malondialdehyde, MDA) and in the activity of antioxidant defense enzymes (superoxide dismutase, SOD; catalase, CAT; ascorbate peroxidase, APX; glutathione reductase, GR). Ca, K, and Ca + K supplementation caused further enhancements in the activity of these enzymes but significantly decreased contents of H_2_O_2_ and MDA, also that of Cd accumulation in shoot and root. The contents of total phenol, flavonoid and mineral elements (S, Mn, Mg, Ca and K) that were also suppressed in Cd stressed plants in both shoot and root were restored to appreciable levels with Ca- and K-supplementation. However, the combination of Ca + K supplementation was more effective in bringing the positive response as compared to individual effect of Ca and K on Cd-exposed *C. arietinum*. Overall, this investigation suggests that application of Ca and/or K can efficiently minimize Cd-toxicity and eventually improve health and yield in *C. arietinum* by the cumulative outcome of the enhanced contents of organic solute, secondary metabolites, mineral elements, and activity of antioxidant defense enzymes.

## Introduction

Cadmium (Cd), a naturally occurring element having no known beneficial role is a highly toxic environmental pollutant (Mobin and Khan, 2007; Shamsi et al., 2008; Asgher et al., 2015). Cd can enter the environment *via* multiple pathways including atmospheric deposition, wastewater irrigation, phosphatic fertilizers, usage of metal-containing pesticides and many industrial processes (Amirjani, 2012; Abdel Latef, 2013). Plants can easily uptake Cd by roots and transport it to shoots, which eventually cause toxicity there (Talukdar, 2012; Abdel Latef, 2013). Accumulation of Cd brings complex changes in plants at physiological, biochemical and genetic levels (El-Beltagi and Mohamed, 2013). The most obvious symptoms are: (i) inhibition of seed germination and suppression of plant growth (Siddique et al., 2012; Abdel Latef, 2013), (ii) degeneration of chlorophyll (Chl) synthesis and disturbance in Calvin cycle enzymes leading to decrease in photosynthesis (Mobin and Khan, 2007; Shamsi et al., 2008), and (iii) induction of stomatal closure due to its effect on the water balance of the plant (Perfus-Barbeoch et al., 2002). Additionally, tissue/organ-Cd-burden can alter carbohydrates, proline (Pro) and protein contents (Siddique et al., 2012; Abdel Latef, 2013; El-Beltagi and Mohamed, 2013; Mondal, 2013), perturb the absorption of nutrients such as N, P, K, Ca, Mg, Mn, Cu, Zn, Fe, and Ni (Sandalio et al., 2001; Siddique et al., 2012; Abdel Latef, 2013), and can also elevate the reactive oxygen species (ROS) levels (Ahmad et al., 2010, 2015). The excessive or non-metabolized ROS can lead to oxidation of organic molecules like lipids (Ahmad et al., 2010, 2015). The lipid peroxidation is generally reflected by increased concentration of malondialdehyde (MDA) content (Abdel Latef, 2013; Ahmad et al., 2015; Anjum et al., 2015).

Plant can sustain cellular ROS-attack through important endogenous protective strategies consisting of enzymatic and non-enzymatic systems (Ahmad et al., 2008, 2010, 2015, 2016a,b; Hasanuzzaman and Fujita, 2011, 2013). Antioxidant defense enzymes like superoxide dismutase (SOD), catalase (CAT), ascorbate peroxidase (APX), and glutathione reductase (GR) have been reported to reduce the concentrations of superoxide (${\text{O}}_{2}^{∙-}$) and hydrogen peroxide (H_2_O_2_) content in plants (Ahmad et al., 2015). Accumulation of protective solutes like Pro and protein in the leaf is a unique plant response to heavy metal stress (El-Beltagi and Mohamed, 2013). Total phenols and flavonoids got induced under low concentrations of metal stress in plants; however, their levels can decline at higher metal concentrations (Cetin et al., 2014). During the last decade, efforts were made to control the level and improve the efficiency of above mentioned defense system components by exogenously supplying mineral elements (Anjum et al., 2012; Nazar et al., 2012) and phytohormones (Khan et al., 2012; Asgher et al., 2015) to the stressed plants.

Calcium (Ca) is a divalent cation and an essential element that plays important role in the structure and permeability of cell membranes, plant cell division and elongation, carbohydrate translocation and N-metabolism (White, 2000; El-Beltagi and Mohamed, 2013). Ca also plays regulatory role in plant cell metabolism, signal transduction and in the absorption of nutrients across cell membranes (Talukdar, 2012; El-Beltagi and Mohamed, 2013). Recent studies showed a major role of Ca in alleviating the inhibitory effects of Cd on growth and physiological processes of plants (Suzuki, 2005; Siddique et al., 2012; El-Beltagi and Mohamed, 2013; Ahmad et al., 2015). Potassium (K) is also an essential macronutrient and the most abundant cation in plant tissues. It consists of up to 10% on the basis of dry weight (Zhao et al., 2003; Siddique et al., 2012). K plays multifarious roles in plant growth and development as it stimulates cell elongation and preserves osmoregulation, and is also involved in stomatal movement, photosynthesis, decrease uptake of Cd, synthesis of soluble carbohydrates, protein, and soluble nitrogen containing compounds (Zhao et al., 2003; Siddique et al., 2012; Zorb et al., 2014). It also acts as a cofactor for many enzymes and activates several enzymes by changing their conformation through the stabilization of pH between 7.0 and 8.0 (Mengel, 2007; Siddique et al., 2012).

Legumes are more sensitive to Cd toxicity than cereals and grasses and thus they can lead to severe suppression of biomass and yield production even under minute quantities of Cd (Shamsi et al., 2008). Chickpea (*Cicer arietinum* L.) is an essential legume crop grown worldwide because it serves as a prime source of proteins for the growing population and it is also used as green manure and fodder for animals. Apart from the proteins, the seeds are also rich in fats and carbohydrates (Rasool et al., 2013, 2015). Insights into *C. arietinum* responses to Cd-exposure, and potential strategies for minimization of Cd-impacts are meager in literature. Additionally, the efficacy of Ca and K in alleviating Cd stress has not been tested in legumes such as *C. arietinum*. So, this work was undertaken to check whether exogenous application of Ca and K can protect *C. arietinum* health and productivity against Cd exposure. Notably, important physio-biochemical parameters, enzymatic activities and the status of organic osmolyte (Pro) and secondary metabolites were assessed to understand potential mechanisms underlying of Cd-tolerance in *C. arietinum*.

## Materials and methods

### Plant culture and exposure conditions

Seeds of chickpea (*Cicer arietinum* L.) were sown in pots containing peat, perlite and sand (1:1:1, v/v/v) under glasshouse conditions. Four-days old germinated *C. arietinum* seedlings were shifted to pots (one plant per pot) supplemented with nutrient solution (200 ml pot^−1^). Seedlings were allowed to grow for one more week at average day/night temperature of 24°C/15°C. The 11-day-old-plants were treated with different concentrations of Cd (CdSO_4_.8H_2_O) dissolved in nutrient solution with or without spray of Ca (CaCl_2_) and K (KCl_2_). Treatments consisted of: C- Nutrient solution alone (control); C + Ca: 0 μM Cd + 50 mM Ca + 0 mM K; C + K: 0 μM Cd + 0 mM Ca + 10 mM K; C + Ca + K: 0 μM Cd + 50 mM Ca + 10 mM K; Cd stress alone: 200 μM Cd + 0 mM Ca + 0 mM K; Cd + Ca: 200 μM Cd + 50 mM Ca + 0 mM K; Cd + K: 200 μM Cd + 0 mM Ca +10 mM K; Cd + Ca + K: 200 μM Cd + 50 mM Ca + 10 mM K.

Cd was applied to pots every week from the first day of treatment (i.e., 11-day-old plants) up to day 30 (41-day-old plants) excluding the control which was supplied with nutrient solution only. CaCl_2_ (50 mM) and KCl_2_ (10 mM) were mixed with Tween-20, and sprayed to plants with a manual sprayer (10 ml plant^−1^) every alternate day, from the 7th day of the treatment-initiation. The Ca and K concentrations were selected based on our preliminary experiments. Each treatment was replicated five times in a randomized block design and each replicate included 5 plants.

## Estimations and bioassays

### Growth and crop yield

At the end of the experiment the plants were taken out of the pots and washed well with tap water. Shoot and root length was measured with a scale. Fresh weight (FW) of shoot and root was measured directly by weighing the fresh samples. For the dry weight (DW), the samples were oven dried for 24 h at 80°C and then weighed. Yield parameters (pods and seeds) were also measured manually.

### Cadmium accumulation

The Cd accumulation in the shoot and root tissues was determined using a Perkin-Elmer (Analyst Model 300) atomic absorption spectrophotometer. The heavy metal content was expressed as μg g^−1^.

### Photosynthetic pigments

The photosynthetic pigments were determined by the method of Hiscox and Israelstam (1979). The absorbances were read at 480, 510, 645, 663 nm by spectrophotometer (Beckman 640 D, USA) with DMSO as blank.

### Proline and protein

The Pro content in leaves was estimated by the method of Bates et al. (1973). The absorbance was measured spectrophotometerically (Beckman 640 D, USA) at 520 nm and toluene was used as blank.

The method of Bradford (1976) was employed for the estimation of protein from leaves. The absorbance was measured at 595 nm by spectrophotometer (Beckman 640 D, USA) with bovine serum albumin as blank.

### Total phenols and flavonoids

The leaf sample was grounded and extracted with methanol at room temperature. The extract containing phenolic content was reduced by Folin-Ciocalteu reagent using the method of Chun et al. (2003). The mg gallic acid equivalent (GAE) g^−1^ of extract (mg g^−1^) expresses the total phenolic content. The flavonoids content was estimated as per colorimetric method described by Zhishen et al. (1999). Catechin was used as standard for the calibration curve. The absorbance was read at 510 nm by spectrophotometer (Beckman 640 D, USA) and flavonoids content was expressed as mg catechin equivalents g^−1^ of extract (mg g^−1^).

### H_2_O_2_ and MDA

H_2_O_2_ in fresh leaves was estimated by the method of Velikova et al. (2000). The absorbance was read at 390 nm by spectrophotometer (Beckman 640 D, USA). For the estimation of MDA content in fresh leaves, the method of Heath and Packer (1968) was employed. The absorbances were recorded at 532 and 600 nm spectrophotometerically (Beckman 640 D, USA) for the calculation of MDA equivalents. Thiobarbituric acid (1%) in 20% trichloroacetic acid was used as control.

### Enzyme extractions and assays

Fresh leaves (0.5 g/sample) were homogenized in presence of 100 mM Tris-HCl (5.0 ml, pH 7.5), 5.0 mM DTT (Dithiothreitol), 10 mM MgCl_2_, 1.0 mM EDTA (Ethylenediaminetetraacetic acid), 5.0 mM magnesium acetate and 1.5% Polyvinylpyrrolidone (PVP)-40. The reaction mixture was supplemented with serine and cysteine proteinase inhibitors [1.0 mM Phenylmethanesulfonyl fluoride (PMSF) + 1.0 μg/ml aproptinin]. The homogenate was centrifuged at 10,000 × g for 15 min (4°C) after the filtration through cheesecloth. The supernatants collected served as sources for determination of SOD (EC 1.15.1.1), CAT (EC 1.11.1.6), and GR (EC 1.6.4.2) activities. For the determination of APX activity, leaf sample was separately grounded in a homogenizing medium containing 2.0 mM ascorbic acid (AsA).

SOD activity was determined by the method of Van Rossum et al. (1997) after the photoreduction of nitrobluetetrazolium (NBT). The absorbance was recorded spectrophotometerically (Beckman 640 D, USA) at 560 nm. One unit of SOD is the quantity of protein that hampers 50% photoreduction of NBT and the activity was expressed as enzyme unit (EU) mg^−1^ protein.

The method of Luck (1974) was employed for the assay of CAT activity. The absorbance was recorded at 240 nm by spectrophotometer (Beckman 640 D, USA) and EU mg^−1^ protein expresses the CAT activity. The method of Nakano and Asada (1981) was used for the assay of APX activity (EC 1.11.1.11). The absorbance was measured spectrophotometerically (Beckman 640 D, USA) at 290 nm and EU mg^−1^ protein expresses the APX activity. One unit of APX is the quantity of protein used to break down 1.0 μmol of substrate per min at 25°C.

The activity of other important enzyme GR was estimated by the method of Carlberg and Mannervik (1985). The absorbance was read at 340 nm by spectrophotometer (Beckman 640 D, USA). GR activity was expressed as μmol NADPH oxidized min^−1^ (EU mg^−1^ protein).

### Nutritional elements

Dried shoot and root samples (0.1 g) were powdered and digested in H_2_SO_4_/HNO_3_ mixture (1/5, v/v) for 24 h, then were treated with HNO_3_/HClO_4_ mixture (5/1, v/v). Atomic absorption spectrophotometer (Analyst 300, Perkin-Elmer, Germany) was used for the estimation of elemental content (S, Mn, Mg, Ca, and K) in both shoot and root.

### Statistical analysis

Statistical analysis was executed utilizing one-way analysis of variance (ANOVA) followed by Duncan's Multiple Range Test (DMRT). The values are mean ± standard error (SE) of five replicates in each group. *P* ≤ 0.05 were considered as significant.

## Results

### Growth, biomass yield, and yield attributes

The length and weight (fresh and dry) of shoot and root and yield attributes in terms of number of pods and seed yield in *C. arietinum* subjected to Ca and/or K under Cd stress and normal conditions are presented in Table 1. Cd stress reduced the shoot length, root length, shoot FW, shoot DW, root FW, root DW, number of pods and seed yield by 58.25, 62.50, 41.26, 21.80, 48.17, 34.84, 49.60, and 52.62%, respectively in comparison with control. Application of Ca and K enhanced the shoot length by 66.76, 39.05, and 95.54% and root length by 34.79, 54.09, and 107.60% with Cd + Ca, Cd + K and Cd + Ca + K, respectively compared to Cd treated plants alone (Table 1). The shoot FW and DW were also elevated by the supplementation of Ca and K and the maximum increase in shoot FW and DW was 30.59 and 20.60%, respectively with Cd + Ca + K treatment compared to plants treated with Cd alone (Table 1). The root FW and DW also increased with addition of Ca and K and the highest increase in root FW (30.98%) and root DW (24.41%) was observed at Cd + Ca + K treatment over the plants treated with Cd alone (Table 1). The supplementation of Ca and K improved the pods by 29.42, 25.05, and 68.51% with Cd + Ca, Cd + K and Cd + Ca + K treatments, respectively relative to plants treated with Cd alone. The seed yield also increased by 52.45% with Cd + Ca, 47.13% with Cd + K and 92.21% with Cd + Ca + K treatments over the Cd treated plants alone (Table 1). Control plants treated with Ca and K showed significant elevation in above parameters compared to control plants alone and the more effective treatment was the combination of Ca + K compared to their individual effect (Table 1).

**Table 1:** **Effect of calcium (Ca) and potassium (K) alone and in combination on shoot and root length (cm plant^−1^), shoot FW and DW (g plant^−1^), root FW and DW (g plant^−1^), number of pods (pod plant^−1^) and seed yield (g plant^−1^) in chickpea (*Cicer arietinum* L.) plants under normal and cadmium (Cd) stress conditions**.

**Growth traits**	**Treatments**							
	**C**	**C + Ca**	**C + K**	**C + Ca + K**	**Cd**	**Cd + Ca**	**Cd + K**	**Cd + Ca + K**
Shoot length	24.72 ± 1.37b	29.15 ± 1.45a	27.44 ± 1.41a	32.27 ± 1.48a	10.32 ± 1.10e	17.21 ± 1.24c	14.35 ± 1.18d	20.18 ± 1.26c
Root length	9.12 ± 1.01c	11.15 ± 1.13b	12.33 ± 1.15b	16.28 ± 1.20a	3.42 ± 0.39g	4.61 ± 0.48f	5.27 ± 0.53e	7.10 ± 0.68d
Shoot FW	6.01 ± 0.68c	6.21 ± 0.75b	6.15 ± 0.72b	6.81 ± 0.81a	3.53 ± 0.41g	3.91 ± 0.46f	4.10 ± 0.51e	4.61 ± 0.57d
Shoot DW	2.11 ± 0.33b	2.25 ± 0.36b	2.29 ± 0.39b	2.62 ± 0.43a	1.65 ± 0.13e	1.72 ± 0.17d	1.78 ± 0.18d	1.99 ± 0.23c
Root FW	4.11 ± 0.45c	4.25 ± 0.47c	4.43 ± 0.53b	4.89 ± 0.59a	2.13 ± 0.27f	2.25 ± 0.34e	2.29 ± 0.31e	2.79 ± 0.41d
Root DW	1.32 ± 0.16b	1.51 ± 0.25a	1.57 ± 0.27a	1.98 ± 0.29a	0.86 ± 0.007e	0.91 ± 0.09d	0.99 ± 0.10d	1.07 ± 0.12c
Number of pods	25.02 ± 1.43c	28.35 ± 1.47b	28.11 ± 1.45b	32.62 ± 1.58a	12.61 ± 1.04f	16.32 ± 1.15e	15.77 ± 1.14e	21.25 ± 1.38d
Seed yield	5.15 ± 0.54c	5.94 ± 0.62b	5.91 ± 0.60b	6.11 ± 0.66a	2.44 ± 0.23g	3.72 ± 0.41f	3.59 ± 0.32e	4.69 ± 0.44d

*Data presented are the means ± standard error (SE) of five independent replications (n = 5). Different letters indicate significant difference (P ≤ 0.05) among the treatments. FW, fresh weight; DW, dry weight*.

### Cadmium accumulation

The accumulation of Cd was about 2.40-fold in root as compared to shoot. Cd stressed plants treated with Ca and K recorded the suppression of Cd accumulation by 69.04, 61.72, and 78.29% with Cd + Ca, Cd + K and Cd + Ca + K, respectively in shoot compared to shoot in Cd treated plants alone. The root Cd accumulation was also reduced by 82.39% with Cd + Ca, 69.76% with Cd + K and 85.87% with Cd + Ca + K treatments relative to plants treated with Cd alone (Table 2).

**Table 2:** **Effect of calcium (Ca) and potassium (K) alone and in combination on cadmium (Cd) content in shoot and root (μmol g^−1^ DW) in chickpea (*Cicer arietinum* L.) seedlings under normal and cadmium (Cd) stress conditions**.

**Traits**	**Treatments**							
	**C**	**C + Ca**	**C + K**	**C + Ca + K**	**Cd**	**Cd + Ca**	**Cd + K**	**Cd + Ca + K**
Shoot Cd	ND	ND	ND	ND	15.57 ± 1.21a	4.82 ± 0.49c	5.96 ± 0.61b	3.38 ± 0.32d
Root Cd	ND	ND	ND	ND	37.31 ± 1.82a	6.57 ± 0.80c	11.28 ± 1.02b	5.27 ± 0.58d

*Data presented are the means ± standard error (SE) of five independent replications (n = 5). Different letters indicate significant difference (P ≤ 0.05) among the treatments. DW, dry weight; ND, not detected*.

### Photosynthetic pigment content

Chl *a*, Chl *b* and total Chl decreased by 48.02, 46.51, and 47.69%, respectively in Cd treated plants relative to control (Table 3). On the other hand, Cd stress significantly increased carotenoids content by 35.89% over the control. However, application of Ca or K alone as well as in combination improved photosynthetic pigments in Cd stressed plants. An increase by 44.30, 69.56, 50.00, and 45.28% in Chl *a*, Chl *b*, total Chl and carotenoids, respectively was observed with treatment Cd + Ca + K compared to the plants treated with Cd alone. Control plants supplemented with Ca and K alone as well as in combination also showed increase in pigment content (Table 3).

**Table 3:** **Effect of calcium (Ca) and potassium (K) alone and in combination on the contents of chlorophyll (Chl) (mg g^−1^ FW), proline (μg g^−1^ FW), protein (mg g^−1^ FW), total phenols (mg gallic acid equivalent (GAE) g^−1^extract) and flavonoids content (mg Catechin g^−1^extract) in chickpea (*Cicer arietinum* L.) leaves under normal and cadmium (Cd) stress conditions**.

**Traits**	**Treatments**							
	**C**	**C + Ca**	**C + K**	**C + Ca + K**	**Cd**	**Cd + Ca**	**Cd + K**	**Cd + Ca + K**
Chl *a*	1.52 ± 0.65c	1.75 ± 0.72b	1.63 ± 0.69b	1.94 ± 0.77a	0.79 ± 0.09g	0.96 ± 0.15e	0.87 ± 0.10f	1.14 ± 0.23d
Chl *b*	0.43 ± 0.06c	0.51 ± 0.07b	0.48 ± 0.07b	0.79 ± 0.12a	0.23 ± 0.02e	0.31 ± 0.03d	0.29 ± 0.03d	0.39 ± 0.05d
Total Chl	1.95 ± 0.75d	2.26 ± 0.88b	2.11 ± 0.81c	2.73 ± 0.99a	1.02 ± 0.17h	1.27 ± 0.28f	1.16 ± 0.25g	1.53 ± 0.66e
Carotenoids	0.39 ± 0.01e	0.45 ± 0.04d	0.41 ± 0.04d	0.49 ± 0.05d	0.53 ± 0.12c	0.69 ± 0.19b	0.57 ± 0.16b	0.77 ± 0.21a
Proline	27.10 ± 1.31g	32.71 ± 1.52f	30.11 ± 1.48f	38.32 ± 1.66e	135.96 ± 3.21d	150.96 ± 3.96b	146.32 ± 3.75c	178.10 ± 3.42a
Protein	18.31 ± 1.15h	24.41 ± 1.35f	21.70 ± 1.30g	29.43 ± 1.44e	37.71 ± 1.65d	48.37 ± 1.72b	44.53 ± 1.68c	57.91 ± 1.83a
Total phenols	4.85 ± 0.56ab	4.93 ± 0.58a	4.92 ± 0.58a	4.99 ± 0.61a	3.01 ± 0.25e	3.76 ± 0.34d	3.64 ± 0.31d	3.92 ± 0.42c
Flavonoids	7.52 ± 0.78c	7.65 ± 0.84b	7.77 ± 0.88b	7.89 ± 0.97a	4.12 ± 0.48f	4.93 ± 0.59e	4.87 ± 0.57e	5.23 ± 0.67d

*Data presented are the means ± standard error (SE) of five independent replications (n = 5). Different letters indicate significant difference (P ≤ 0.05) among the treatments. FW, fresh weight*.

### Proline and protein contents

The results related to the effect of Ca and K on Pro and protein content in Cd stressed chickpea plants are presented in Table 3. The Pro and protein contents increased by 5- and 2-fold, respectively in Cd stressed plants relative to control. Supplementation of Ca and K showed further accumulation in Pro and protein content and the maximum increase in Pro (30.99%) and protein (53.57%) was recorded in plants treated with Cd + Ca + K treatment over the plants treated with Cd alone. Application of Ca and K in combination was more effective in control as well as in treated plants (Table 3).

### Total phenols and flavonoids

The total phenols and flavonoids contents were declined by 37.93 and 45.21% in Cd stressed plants comparison to control plants (Table 3). However, supplementation of Ca and K increased the total phenols by 24.91, 20.93, and 30.23% with Cd + Ca, Cd + K and Cd +Ca + K treatments, respectively as compared to plants treated with Cd alone. Insignificant increase in total phenols was observed by the application of Ca and K individually as well as in combination to control plants (Table 3). Flavonoids content also enhanced by 19.66% with Cd + Ca, 18.20% with Cd + K and 26.94% with Cd + Ca + K treatments relative to Cd treated plants alone (Table 3). Ca and K in combination were proved to me more beneficial as compared to their individual effect in both control and Cd treated plants. Significant increase in flavonoids content was observed in plants supplied with Ca and K individually as well as in combination (Table 3).

### H_2_O_2_ and MDA

**Figure 1:**
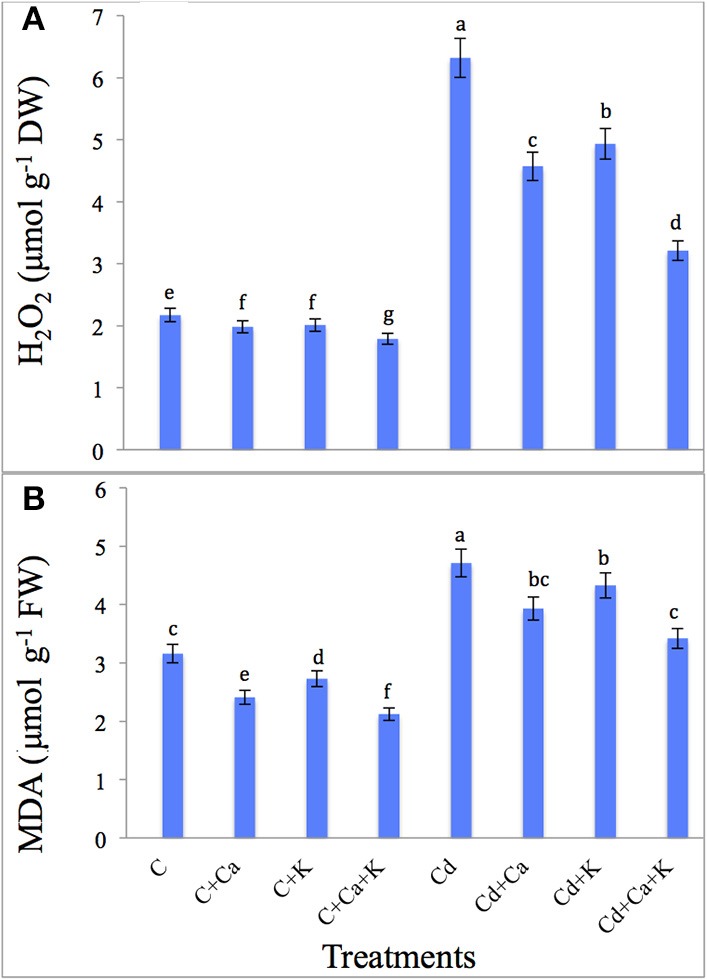
**Effect of calcium (Ca) and potassium (K) alone and in combination on H_2_O_2_ (A), and lipid peroxidation (MDA) (B) in chickpea seedlings under cadmium (Cd) stress**. Data presented are the means ± SE (*n* = 5). Different letters indicate significant difference (*P* ≤ 0.05) among the treatments.

Cd treatment led to a sharp accumulation of H_2_O_2_ (191.24%) and MDA (49.05%) over the control plants. Addition of Ca and K to Cd treated plants reduced the H_2_O_2_ accumulation by 27.68% with Cd+Ca, 21.99% with Cd + K and 49.20% with Cd + Ca + K treatments compared to plants treated with Cd alone. The MDA content also decreased by 16.56, 8.06, and 27.39% with Cd + Ca, Cd + K and Cd + Ca + K, respectively relative to Cd treated plants alone (Figures 1A,B).

### Activities of antioxidant enzymes

**Figure 2:**
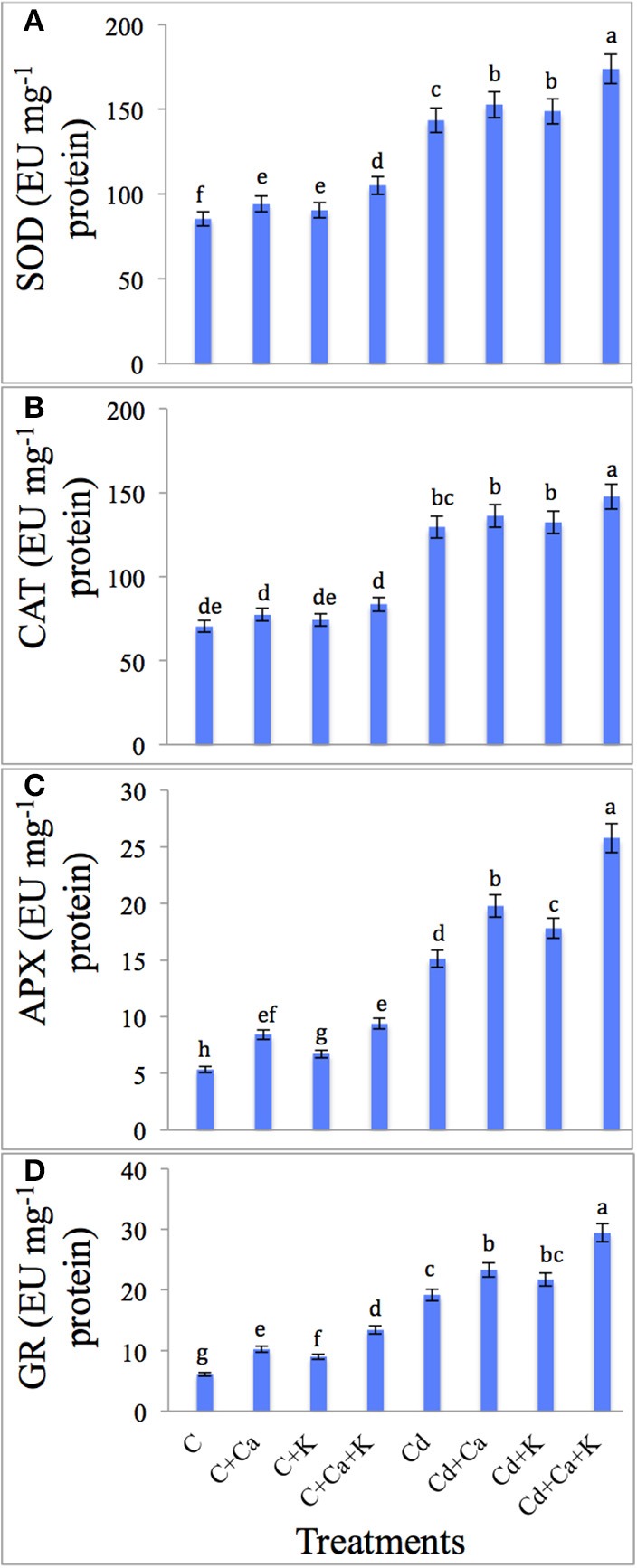
**Effect of calcium (Ca) and potassium (K) alone and in combination on the activity of superoxide dismutase (SOD) (A), catalase (CAT) (B), ascorbate peroxidase (APX) (C), and glutathione reductase (GR) (D) in chickpea seedlings under cadmium (Cd) stress**. Data presented are the means ± SE (*n* = 5). Different letters indicate significant difference (*P* ≤ 0.05) among the treatments.

Cd stress boosted the SOD, CAT, APX, and GR activity by 67.99, 83.90, 183.67, and 215.81%, respectively as compared to control plants. Cd treated plants supplemented with Ca and K further enhanced the activities of SOD by 21.08%, CAT by 14.03%, APX by 70.43% and GR by 53.52% with Cd + Ca + K treatments over the plants treated with Cd alone (Figures 2A–D). Control plants supplemented with Ca and K individually as well as in combination also boosted antioxidant activity in untreated plants (Figures 2A–D).

### Mineral elements

Cd stress significantly affected the pools of mineral nutrients in *C. arietinum* shoot and root. S, Mn, Mg, Ca and K contents in shoot were declined by 36.74, 64.04, 47.14, 33.84, and 49.20%, respectively with Cd treatment under the control plants. The root mineral elements also showed decline by 48.21, 16.52, 18.36, 14.15, and 37.22% in S, Mn, Mg, Ca, and K, respectively with Cd treatment relative to control plants. The application of Ca and K individually as well as in combination improved the mineral elements in both shoot and root under Cd toxicity. The combined effect of Ca + K proved to be more beneficial in comparison to individual effects of Ca and K in restoring the mineral nutrients in shoot and root in control as well as in Cd treated plants (Table 4).

**Table 4:** **Effect of calcium (Ca) and potassium (K) alone and in combination on mineral elements content in shoot and root (μg g^−1^ DW) of chickpea (*Cicer arietinum* L.) plants under normal and cadmium (Cd) stress conditions**.

**Mineral Elements**	**Treatments**							
	**C**	**C + Ca**	**C + K**	**C + Ca + K**	**Cd**	**Cd + Ca**	**Cd + K**	**Cd + Ca + K**
Shoot S	127 ± 3.27c	133 ± 3.35b	129 ± 3.30c	137 ± 3.49a	80.33 ± 2.45f	89.29 ± 2.59e	85.15 ± 2.54e	98.72 ± 2.84d
Shoot Mn	31.21 ± 1.49c	37.05 ± 1.54b	35.19 ± 1.52b	40.32 ± 1.66a	11.22 ± 1.06f	15.71 ± 1.37e	15.32 ± 1.31e	21.81 ± 1.42d
Shoot Mg	275 ± 4.91c	281 ± 4.98b	275 ± 4.90c	288 ± 5.10a	145.34 ± 3.52f	185.57 ± 3.88e	181.12 ± 3.74e	199.33 ± 4.01d
Shoot Ca	132 ± 3.52d	141 ± 3.64b	137 ± 3.58c	144 ± 3.71a	87.32 ± 2.62h	103 ± 3.05f	95.77 ± 2.87g	115.31 ± 3.14e
Shoot K	691 ± 8.10d	703 ± 8.20c	715 ± 8.31b	725 ± 8.37a	351 ± 5.57h	361 ± 5.65g	475 ± 5.80f	502 ± 5.97e
Root S	421 ± 6.21d	430 ± 6.35b	427 ± 6.29c	438 ± 6.44a	218 ± 4.12h	328 ± 5.16f	315 ± 4.98g	385 ± 5.62e
Root Mn	121 ± 3.11c	127 ± 3.33b	125 ± 3.30b	132 ± 3.51a	101 ± 2.95e	110 ± 3.09d	108 ± 3.04d	117 ± 3.15c
Root Mg	855 ± 9.41b	857 ± 9.45b	855 ± 9.42b	860 ± 9.52a	698 ± 7.67f	761 ± 7.89d	752 ± 7.75e	799 ± 8.06c
Root Ca	212 ± 4.31d	221 ± 4.47b	217 ± 4.36c	231 ± 4.59a	182 ± 3.71g	205 ± 4.09e	193 ± 3.91f	211 ± 4.27d
Root K	1370 ± 13.31c	1391 ± 13.45c	1430 ± 13.87b	1475 ± 14.12a	860 ± 9.51g	1075 ± 11.25f	1163 ± 12.09e	1207 ± 12.47d

*Data presented are the means ± standard error (SE) of five independent replications (n = 5). Different letters indicate significant difference (P ≤ 0.05) among the treatments. DW, dry weight*.

## Discussion

**Figure 3:**
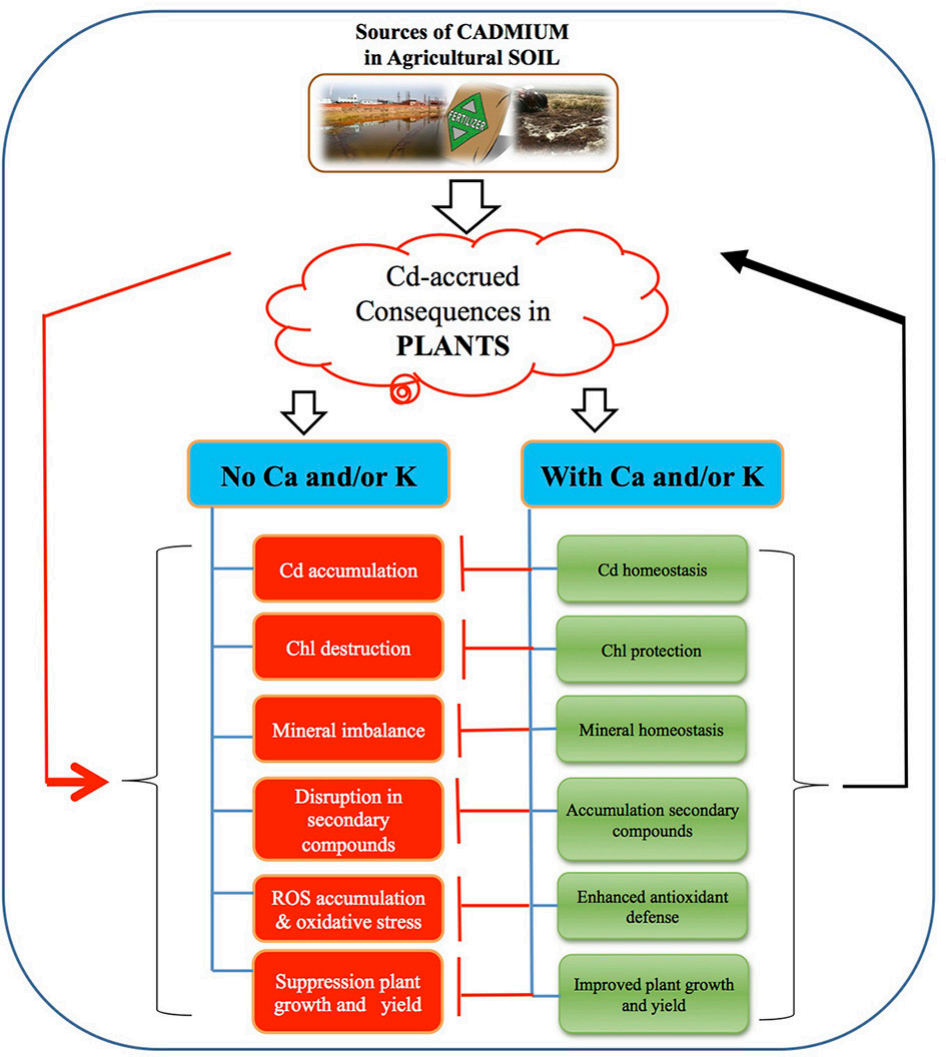
**A possible model showing the strategy of calcium (Ca) and/or potassium (K) induced cadmium tolerance in chickpea**. Exposure of *C. arietinum* to Cd caused an increased in uptake and accumulation of Cd in plant cells. Elevated cellular-Cd induced chlorophyll (Chl)-destruction, ionic disorder, disruption in secondary metabolites, generation of reactive oxygen species (ROS), leading to suppression of plant growth and yield. On the other side, supplementation of Ca and or K resulted into mineral nutrient homeostasis, increased Chl content, accumulation of secondary compounds and higher antioxidant capacity all of which contributed to the mitigation of Cd-induced damage, leading to improved plant growth and yield.

Cd is a non-essential element and its accumulation hampers the growth and development of the plant (Abdel Latef, 2013; Ahmad et al., 2015; Asgher et al., 2015). The suppression in growth under Cd toxicity may be attributed to restricted water and nutritional uptake by plant roots (Dinakar et al., 2009), disturbance in photosynthesis (Dong et al., 2005), reduction of cell wall components and changes in carbohydrates metabolism (Abdel Latef, 2013; Hussain et al., 2013). Our results are in harmony with Mondal (2013) who reported that the reduction of root growth may be due to direct interference of Cd with some hydrolytic enzymes, which plays a pivotal role in transporting the food to the primary root and shoot. He also reported that the reduction in the shoot length may be due to the direct inhibition of cell elongation or cell division, retarded root growth and low nutrients/water transport to the upper parts of shoot (Mondal, 2013). Foliar application of Ca and K alone as well as in combination mitigated the negative impact of Cd toxicity and enhanced the plant growth and yield attributes of chickpea plants (Figure 3), indicating the direct or indirect role of both elements in improving plant growth and biomass yield even under heavy metal stress. Ca may favor cell elongation and cell expansion, finally enhancing the plant growth and was also reported by Hernandez and Almansa (2002) in pea and Abdel Latef (2011a) in canola. This finding is in consistence with Ismail (2008) who reported that supplementation of Ca mitigated the growth inhibition induced by Cd toxicity in common bean. Our previous work on mustard also confirmed that Ca alleviated the Cd induced suppression in growth and biomass yield (Ahmad et al., 2015). Eller and Brix (2016) also proved that shoot and root biomass was enhanced by addition of Ca to Cd stressed *Brassica juncea* plants. K plays a vital role in nullifying the harmful effects of stress on plants as it serves as a catalyst for various enzymatic processes that are essential for plant growth and development (Cakmak, 2005). Deficiency of K in plants has been reported to decrease the rate of net photosynthesis and ribulose-1, 5-bisphosphate carboxylase activity (Cakmak, 2005; Weng et al., 2007). Song et al. (2015) have reported that application of K restricted the fresh weight of peach (*Prunus persica*) seedlings under Zn-toxicity. According to the present study Ca and K in combination proved to be more effective than individual Ca and K and the results are in conformity with the finding of Siddique et al. (2012) in *Vicia faba* under Cd toxicity.

Cd decreased the number of pods and seed yield which coincides with the findings of Siddhu and Khan (2012) in *Phaseolus mungo*. Fatoba et al. (2012) also reported the decrease in yield parameters with increasing concentration of Cd in *Arachis hypogaea* and *Glycine max*. The negative effects of Cd stress on yield attributes have also been reported by Kumar and Dhingra (2005) in mungbean and Ali Khan and Siddhu (2006) in *Vigna mungo*. Application of Ca and K enhanced the yield parameters under Cd stress in the current study that may be attributed to (i) increased mineral and water uptake, (ii) restoration of photosynthetic pigments and rehabilitation of other metabolic processes affected by Cd stress.

The accumulation of Cd was more in root compared to shoot and the results coincided with previous reports on different plant species (Amirjani, 2012; Mondal, 2013). The root is first entry point for immobilization of Cd by means of the cell wall and extracellular carbohydrates (Amirjani, 2012). The lower accumulation of Cd in shoot may be due to the retention of Cd ions in the root and preventing the transport of excess Cd to the aerial parts of *C. arietinum*. The application of Ca and K individually or in combination reduced accumulation of Cd in both root and shoot in the present study (Figure 3). The protection provided by Ca for alleviating Cd toxicity can be due to various mechanisms like (i) the displacement of cell-surface toxic cations by Ca reduced the negativity of plasma membrane and mitigation the deleterious effect of cationic toxicants (Kinraide, 1998) (ii) elimination of anxious Cd by the formation and exudation of Cd/Ca containing crystals from the trichome head cells (Choi et al., 2001) (iii) Cd uptake via Ca channels blockers, diltiazem, verapmil, nifedipine and nitrendipine (Hinkle et al., 1987) (iv) competition for metal ion influx (Suzuki, 2005), and (v) complexing Cd with phytochelatins such as metalothionin and cysteine-rich proteins (Suzuki et al., 2002; Song et al., 2004). The impact of K fertilizers on plant accumulation of Cd has rarely been studied in detail earlier. Availability of K minimizes the uptake of Cd as both ions (Cd and K) are in competition for the same transmembrane carrier (Clarkson and Luttge, 1989; Rivetta et al., 1997). Noraho and Gaur (1995) also reported that K application resulted in less Cd uptake in *Lemna polyrrhiza*. The molecular mechanism how K decreases uptake of Cd needs further elucidation.

Our data regarding the photosynthetic pigments (Chl *a, b*, and total Chl) in *C. arietinum* showed decline with Cd stress. This decline may be due to (i) inhibition of photosynthetic electron transport chain (Siddique et al., 2012), (ii) inhibition of the sulfhydryl requiring enzymes of Chl biosynthesis (Siddique et al., 2012), (iii) damage of PS II reaction center in the leaf (Mondal, 2013), (iv) replacement the central Mg^2+^ of Chl in plants to prevent light harvesting (Abdel Latef, 2013), (v) reduction the uptake of P which contributes to pigment biosynthesis (Abdel Latef, 2013) (vi) induction of lipid peroxidation, which causes degradation of photosynthetic pigments (Abdel Latef, 2013; Ahmad et al., 2015). Carotenoids are categorized in lipophilic antioxidant group and have the ability to scavenge various forms of ROS such as singlet oxygen (^1^O_2_) (Mourato et al., 2012). Carotenoids act as precursors to signaling molecules that have a positive impact on plant growth and development and responses under biotic and abiotic stress (El-Beltagi and Mohamed, 2013). Interestingly, in our study, the synthesis of carotenoids decreased the oxidative damage induced by Cd toxicity and is also reported by El-Beltagi and Mohamed (2013) in *Pisum sativum* and Abdel Latef and Abu Alhmad (2013) in broad bean with copper stress. Supplementing the medium with Ca enhanced photosynthetic pigments in *Vicia faba* (Siddique et al., 2012) and *Brassica juncea* (Ahmad et al., 2015) under Cd toxicity. Ca served as secondary messenger for cytokinin action in improving synthesis of Chl (Lechowski and Bialczyk, 1993). K also plays a remarkable role in development of photosynthetic pigments by preventing degradation of newly formed Chl and aminolevulinic acid (ALA) (Siddique et al., 2012). Song et al. (2015) also reported that application of K enhanced the photosynthetic efficiency by increasing chlorophyll content in zinc stressed *P. persica*. Thus, the improvements in photosynthetic pigments are due to the combined effect of Ca and K that provides tolerance to *C. arietinum* plants against Cd stress (Figure 3).

Pro accumulation under Cd exposure appears to be prevalent among plants (Zhao, 2011; Siddique et al., 2012; El-Beltagi and Mohamed, 2013). Pro plays a pivotal role in protection of plants from abiotic and biotic stresses (Ahmad et al., 2010, 2011, 2012a,b, 2014, 2015; Siddique et al., 2012; Abdel Latef and Tran, 2016). The possible roles of Pro in heavy metals toxicity can be due to (i) its ability to balance the plant water potential (Fariduddin et al., 2009), (ii) its ability to save enzymes from inhibition (Sharma and Dietz, 2006), (iii) its function as metal chelator (Sharma et al., 1998), and (v) its antioxidant property (Zhao, 2011). The accumulation of protein during heavy metal stress may furnish a reserve form of N that could be re-utilized later and helps in osmoregulation. The greater increase in protein content under Cd stress was followed by a significant decline in growth. Thus, it may be suggested that under these conditions, *C. arietinum* transfer most of the synthesized protein from a state of growth to a state of osmoregulation (survival). Thus, *C. arietinum* under Cd stress synthesized the protein for survival rather than for growth. However, application of Ca and K together was found to be efficient for augmenting further accumulation of Pro and protein under Cd stress. Ca signaling is involved in Probiosynthesis (Verslues and Sharma, 2010). Calmodulin, a Ca signaling unit has been reported to activate MYB (myeloblastosis) transcription factor which further activates different downstream genes including Δ^1^- pyrroline-5-carboxlyate synthetase 1 (*P5CS1*) (Yoo et al., 2005). Phospholipase “C” is another calcium signaling component which upregulated the expression of *P5CS1* gene in Arabidopsis under salt stress (Parre et al., 2007). The Pro biosynthesis occurs through the activation of the precursors like glutemic acid, arginine and ornithine (Stewart and Bogess, 1977) and N-acetyl gutamic acid (Morris et al., 1969). K has the ability to interact with arginine in synergistic way enhancing the Pro content (Nageswararao et al., 1981). The accumulation of protein by application of Ca in the present study is in accordance with Abdel Latef (2011a) in canola plants under seawater stress. Recently, Yousuf et al. (2015) also reported that, application of K and Ca fertilizers regained the protein content in salt-stressed Indian mustard. K supplementation also enhanced the protein content in salt stressed wheat plants (Zheng et al., 2008). K has a leading role in plant protein synthesis (Blevins, 1985). The role of Ca and K in enhancing protein content may also be attributed to enhanced protein synthesis, decreased proteolysis, lowering of enzyme denaturation that were disturbed during abiotic stress (Levitt, 1980).

The decrease in total phenols and flavonoids content subjected to Cd stress is corroborated with the findings of Kapoor et al. (2014). Lachman et al. (2005) also reported the decrease in flavonoids content with increasing concentration of Cd in barley. Bai et al. (2004) reported that flavonoids play a great role in metal chelation. The increase in Cd content decreased the free flavonoids content in the present study. Low concentration of Cd stress increases the flavonoids content in many plants; however, at higher concentrations a decrease was observed (Cetin et al., 2014) as also observed in present study. Phenolic compounds played a key role in protecting the plants against toxic effects induced by different stresses. Total phenols and flavonoids have the antioxidant property because they are electron-donating agents (Michalak, 2006), thus scavenge ROS. Phenolic compounds are synthesized quickly after stress exposure through the signaling processes (Bais et al., 2002; Li et al., 2005). Application of Ca and K increased the total phenols and flavonoids content under Cd stress (Figure 3). The mechanism underlying previous process is still unclear. However, Ca and K-mediated increase the total phenols and flavonoids can be possible since: (i) Ca and K hamper the uptake of Cd as described above, (ii) Ca has a role in signaling and helps in upregulation of respective genes for polyphenols biosynthesis (Xu et al., 2014), and (iii) the reduced Cd in the cells has the ability to stimulate the phenylalanine ammonia lyase (PAL) enzyme activity, an important enzyme in phenylpropanoid biosynthesis (Kuthanova et al., 2004).

H_2_O_2_ production is very harmful for many biochemical reactions; however, it also plays a signaling role in plant growth and development (Mazid et al., 2011). H_2_O_2_ also interferes with the Calvin cycle that finally leads to decreased rate of photosynthesis (Hussain et al., 2013) and is usually proclaimed stress criterion in plants (Mourato et al., 2012). Cd stress causes lipid peroxidation and is a result of oxidative stress (Abdel Latef, 2013). MDA is the end product of peroxidation of lipid membranes and its concentration is ordinarily considered as a general criterion of lipid peroxidation as well as the stress level (Shamsi et al., 2008; Abdel Latef, 2011a, 2013; Ahmad et al., 2015; Anjum et al., 2015). Cd stress induced the production of ROS resulting in malfunctioning of biomolecules (Ahmad et al., 2015, 2016a). Cd-induced loss of membrane permeability, coupled with increased MDA production, has also been observed by Abdel Latef (2013) in pepper (*Capsicum annuum*) and Hussain et al. (2013) in *Zea mays* seedlings. Our results are also in agreement with findings of Li et al. (2016) who reported Cd-induced oxidative stress via an enhanced production of H_2_O_2_ and MDA contents in *Arabidopsis*. However, the Cd stressed plants that were subjected to Ca and K alone as well as in combination, showed less accumulation of H_2_O_2_ and MDA content (Figure 3). This might be due to the activity of antioxidant enzymes and Pro accumulation which have an adaptive significance, because of their role in detoxification of free radicals thus reducing oxidative damage to membranes under Cd stress (Siddique et al., 2012; Li et al., 2016). Talukdar (2012) reported that mitigation of Cd-induced stress by Ca application was due to decline in the levels of H_2_O_2_ accumulation and consequently protects the membranes from oxidative damage. Li et al. (2016) reported that Ca protection against Cd-induced oxidative injuries is achieved through avoidance of H_2_O_2_ generation as well as the reduction of Cd uptake. Ca binds to the membrane phospholoipids thus stabilizing the lipid bilayer and providing the structural integrity (Hirschi, 2004; Yousuf et al., 2015) and is exhibited by the reduced MDA content in the plants treated with Ca (Siddique et al., 2012; Talukdar, 2012; Li et al., 2016). K plays an active role in reducing ROS production by decreasing the activity of NAD(P)H oxidases and maintaining photosynthetic electron transport (Cakmak, 2005; Siddique et al., 2012).

Plants synthesize numerous antioxidant enzymes as defense mechanism to cope with the oxidative stress (Foyer and Shigeoka, 2011). The antioxidant enzymes such as SOD, CAT, APX and GR are important components for preventing the oxidative stress in plants (Li et al., 2016). SOD is the first line of defense that catalyzes the dismutation of ${\text{O}}_{2}^{∙-}$ radicals to H_2_O_2_ and O_2_ (Alscher et al., 2002; Mourato et al., 2012). H_2_O_2_ is also toxic to plant cells and CAT carries out its decomposition to water and oxygen (Ahmad et al., 2015). APX showed much attraction toward H_2_O_2_ as compared to CAT, advocating that they have different roles in ROS detoxification. CAT is accountable for the elimination of surplus H_2_O_2_ and APX is labile for maintaining lower concentrations of H_2_O_2_ (Mittler, 2002; Mourato et al., 2012). GR is important in maintaining the pool of reduced glutathione by H_2_O_2_ removal and by activating the ascorbate-glutathione cycle (Asada, 1999; Abdel Latef, 2011b). In this work, antioxidant enzymes (SOD, CAT, APX, and GR) increased markedly under Cd stress. The increase in antioxidant enzyme activity might be due to the overproduction of H_2_O_2_, which would produce oxidative damages in Cd stressed plants (Li et al., 2016). The elevation in the activities of these enzymes showed that *C. arietinum* had the ability to acclimate Cd stress by developing an antioxidant defense system. In our work, GR was stimulated more than other antioxidant enzymes under Cd stress suggesting that GR is a major player in H_2_O_2_-scavenging in Cd stressed plants. An elevated activities of antioxidant enzymes and lower hydrogen peroxide and lipid peroxidation with Ca application suggested that Ca prevented the structural and functional deterioration of cell membranes (Abdel Latef, 2011a; Ahmad et al., 2015; Figure 3). Recent studies reported that application of Ca was found to be effective in enhancing the tolerance of plant to Cd stress by enhancing antioxidant systems (Siddique et al., 2012; Talukdar, 2012; El-Beltagi and Mohamed, 2013). The enhanced activity of antioxidant enzymes may also be due to the active role of K, which activates more than 60 enzyme systems which aid in photosynthesis, regulate stomatal opening and nutrient-translocation, and enhancement in starch synthesis (Shamsi et al., 2008). Under stress, K also plays great role in protein synthesis, e.g., thioredoxin, glutaredoxin, cyclophilin which facilitate regeneration of the reduced form of peroxiredoxins participate in reducing the ROS formation in plants under biotic and abiotic stress (Tripathi et al., 2009; Siddique et al., 2012). Increases in the activities of all four-enzymes led to decrease in the level of both H_2_O_2_ and MDA content in response to application of Ca and K. Consequently, this ensured recovery of plant growth by mitigating the oxidative damage induced by Cd treatment in chickpea plants (Figure 3).

Increased Cd concentration also decreased the uptake of mineral elements as reported by Gonçalves et al. (2009). The plant nutrients and Cd ions can compete for the same transporters; therefore, presence of Cd can result in mineral nutrients deficiency (Nazar et al., 2012; Figure 3). Rivelli et al. (2014) also reported that increasing concentration of Cd decreased the essential mineral uptake in sunflower. Exogenous application of Ca and K alleviated the negative effect of Cd on these nutrients in the present study (Figures 2A–D). Application of Ca and K might have decreased the Cd-competition for the same transporter. Ca also blocks the entry of Cd in root through Ca channels, and controls the translocation of root-Cd to leaves. Thus, applied Ca to Cd-exposed plants can restore their transpiration and photosynthesis (Hayakawa et al., 2011).

## Conclusions

It is inferred from the results of present work that exogenous application of Ca and/or K mitigated the negative effects of Cd on *C. arietinum* through osmoregulation, by adjusting the membrane stability, photosynthetic pigments and lowering H_2_O_2_ and MDA contents. Further, protection under Cd stress was achieved *via* improved activities of antioxidant enzymes such as SOD, CAT, APX, and GR. Supplementation of Ca and K improved the secondary metabolites that were suppressed by Cd-exposure. The study also confirmed Cd-accrued decreases in the uptake of essential mineral elements and the crop yield. Contrarily, exogenously applied Ca and K improved the crop yield through the enhancing uptake of mineral elements and the biosynthesis of photosynthetic pigments. Thus, application of mineral nutrients could be a strategy to improve the growth, development and economic yield in plants/crops growing in toxic metal-polluted soils.

## Author contributions

PA, AA, EA, and AH designed and performed the experimental work. PA, AA, EA, and AH have written the manuscript. MS, NAA and SG contributed in discussion part. MS and SG have done the statistical analysis, and formatting of the paper.

### Conflict of interest statement

The authors declare that the research was conducted in the absence of any commercial or financial relationships that could be construed as a potential conflict of interest.
